# Real-World Effectiveness, Safety, and 36-Month Persistence of Upadacitinib in Elderly Patients with Moderate-to-Severe Atopic Dermatitis: The 3–6 Month Critical Window and the Role of Early Itch Response and Psychotropic Medication Use

**DOI:** 10.3390/jcm15176593

**Published:** 2026-08-26

**Authors:** Yaoyao Wang, Xianzhen Chen, Yongdong Wang, Jiang Zhu, Boya Zhang, Defeng Kong, Yudan Tian, Tianze Yu, Haiyan Yu, Kejian Zhu, Hao Cheng

**Affiliations:** Department of Dermatology, Sir Run Run Shaw Hospital, Zhejiang University School of Medicine, 3 East Qingchun Road, Hangzhou 310016, China; wangyaoyaochn@zju.edu.cn (Y.W.);

**Keywords:** atopic dermatitis, elderly, upadacitinib, JAK inhibitor, drug persistence, real-world evidence, early itch response, psychotropic medication

## Abstract

**Background/Objectives**: Elderly patients (≥65 years) with atopic dermatitis (AD) often carry multimorbidity and face elevated risks with Janus kinase (JAK) inhibitors. Phase III trials included few older adults, and real-world data beyond 12 months remain scarce. We evaluated 36-month efficacy, safety, and drug persistence of upadacitinib 15 mg once daily in 62 Chinese elderly patients. **Methods**: This retrospective cohort study was conducted at Sir Run Run Shaw Hospital (March 2022-July 2026). All patients received upadacitinib 15 mg once daily. Assessments included Week 4 pruritus Numerical Rating Scale (NRS) response, Eczema Area and Severity Index (EASI) 75/90, and Investigator’s Global Assessment (IGA) 0/1 at Weeks 12 and 24. Drug persistence was estimated with the Kaplan–Meier method (36-month cutoff), and predictors were identified by Cox regression. **Results**: Median age was 70.0 years, 75.8% were male, and baseline EASI was 34.5, with substantial comorbidity (Charlson Comorbidity Index [CCI] 5.0; psychotropic use 46.8%). Week 4 itch response was 70.8%. EASI 75 reached 75.0% (IGA 0/1 44.4%) at Week 12, rising to 88.0% (64.0%) at Week 24. Persistence declined from 88.7% at Month 3 to 66.1% at Month 6, the steepest drop, reaching 38.7% at Month 36. Treatment-emergent adverse events (TEAEs) occurred in 54.8% and serious adverse events (SAEs) in 8.1%, with herpesvirus infection (21.0%) being the most common event, and there were no adverse event (AE)-related discontinuations, major adverse cardiovascular events (MACE), or venous thromboembolism (VTE). In multivariate analysis, Week 4 itch response predicted longer persistence (hazard ratio [*HR*] = 0.203, 95% confidence interval [*CI*]: 0.091-0.452, *p* < 0.001), while psychotropic medication use predicted shorter persistence (*HR* = 2.814, 95% *CI*: 1.237-6.403, *p* = 0.014). **Conclusions**: Upadacitinib showed robust efficacy and acceptable safety over 36 months in elderly AD patients. Persistence dropped most sharply between Months 3 and 6. Week 4 itch response and psychotropic medication use independently predicted persistence. A structured Month 3 visit integrating itch reassessment, mental health screening, and dose optimization may improve long-term outcomes.

## 1. Introduction

Elderly patients (≥65 years) with moderate-to-severe atopic dermatitis (AD) endure persistent pruritus, sleep disruption, psychosocial comorbidity, and marked quality-of-life impairment [1]. Treatment in this group is especially challenging: conventional agents such as cyclosporine and methotrexate are limited by hepatotoxicity, nephrotoxicity, and drug–drug interactions in those already taking multiple medications, while biologics including dupilumab are not universally effective, with only up to one-third of patients achieving complete skin clearance [1]. Many older patients respond inadequately to these treatments or cannot tolerate them (e.g., due to conjunctivitis or injection-site reactions) [1]. A proportion of patients therefore need alternative systemic options.

Upadacitinib, an oral selective JAK inhibitor, produced rapid itch relief and robust skin clearance in phase III trials [2,3]. These trials, however, enrolled few patients aged 65 years or older and systematically excluded those with significant comorbidities. Short-term real-world data in older adults are beginning to accumulate. The Italian IL AD study followed 72 patients aged over 60 years for 52 weeks and reported acceptable efficacy with no major adverse cardiovascular events (MACE) or venous thromboembolism (VTE) [4]. The Western Japan Atopic Dermatitis Registry (WJADR) reported similar safety outcomes in an Asian population that included many elderly patients [5]. Despite these contributions, follow-up in published cohorts rarely exceeds 12 months, and long-term drug persistence specifically in those aged 65 years and older remains largely unknown [6,7]. This evidence gap is compounded by the FDA class-wide black box warning for JAK inhibitors, which cites risks of MACE, VTE, and malignancy [8], and has further amplified prescribing caution for older patients whose baseline cardiovascular risk is already elevated.

Beyond regulatory cautions, the long-term determinants of JAK inhibitor persistence in older patients remain poorly understood. We addressed this question in two steps. First, persistence was stratified by four baseline characteristics selected to capture distinct dimensions of geriatric health: Charlson Comorbidity Index (CCI) (somatic disease burden), polypharmacy (treatment complexity), atopic comorbidities (allergic diathesis), and psychotropic medication use (psychological vulnerability) [1,9]. Second, eight candidate variables were entered into Cox regression to identify independent predictors of discontinuation. Among these, two merit particular attention. The first is early itch response. Measurable by Week 4, it is the earliest objective signal of treatment benefit and can help a patient build confidence in continuing therapy. The second is baseline psychotropic medication use. An objective marker of psychological vulnerability, it flags individuals who are more sensitive to treatment risks and whose commitment to therapy is more easily shaken, which makes them particularly susceptible to discontinuation in the face of a black box warning. Neither early itch response nor psychotropic medication use has been examined as a predictor of JAK inhibitor persistence in geriatric AD patients.

To fill these gaps, we retrospectively analyzed 62 Chinese patients aged 65 years or older with moderate-to-severe AD for up to 36 months after initiation of upadacitinib 15 mg once daily. Our objectives were to assess long-term efficacy and overall safety, to describe the trajectory of drug persistence, and to determine which of the candidate variables independently predicted treatment discontinuation.

## 2. Materials and Methods

### 2.1. Study Design and Participants

We conducted a retrospective study of consecutive patients aged ≥65 years who started upadacitinib 15 mg once daily for moderate-to-severe atopic dermatitis (AD) at Sir Run Run Shaw Hospital, Zhejiang University, from March 2022 to July 2026. Eligible patients were identified retrospectively through a systematic search of the electronic medical record (EMR) system at our hospital. AD was diagnosed using the Hanifin and Rajka criteria [1], with secondary confirmation by the Chinese Criteria for Atopic Dermatitis (CCAD) [10]; the Williams UK Working Party criteria were not applied due to low sensitivity in late-onset elderly AD. Patients were included if they had a baseline Eczema Area and Severity Index (EASI) ≥ 16, an Investigator’s Global Assessment (IGA) score ≥ 3, and at least one follow-up visit. We excluded patients with active infection, contraindications to Janus kinase (JAK) inhibitors, concurrent use of other systemic immunosuppressants, or ongoing systemic corticosteroid treatment. Active infection was defined as acute uncontrolled infections (e.g., active tuberculosis, hepatitis B/C, or severe soft tissue infection). Contraindications to JAK inhibitors were defined per standard local guidelines, including severe cytopenias (absolute neutrophil count < 1000/mm^3^, absolute lymphocyte count < 500/mm^3^), severe hepatic impairment, and active infections. The Institutional Review Board of Sir Run Run Shaw Hospital, Zhejiang University approved the study (protocol 20260296, 15 April 2026), which followed the Declaration of Helsinki. Informed consent was waived because of the retrospective design and data anonymization. The study period (March 2022 to July 2026) represents the time frame of patients’ routine clinical visits. Because this was a retrospective analysis of data already recorded in the EMR for routine clinical care, the data extraction and analysis for this study were performed after IRB approval (15 April 2026). Patients were not prospectively recruited or intervened upon during the May–July 2026 period; only their existing routine clinical records were extracted. This study was a purely retrospective analysis of routine clinical data.

### 2.2. Assessments and Definitions

The primary early efficacy and predictor variable was the Week 4 itch response, defined as a ≥4-point reduction in peak pruritus Numerical Rating Scale (NRS) from baseline. Additional efficacy endpoints assessed at Weeks 12 and 24 included EASI 75/90 and IGA 0/1. Comorbidity burden was quantified using the Charlson Comorbidity Index (CCI), calculated according to the updated methodology [11]. Polypharmacy was defined as the regular use of five or more oral medications. Baseline psychotropic medication use was defined as a documented prescription of an antidepressant, anxiolytic, or hypnotic. Atopic comorbidities comprised allergic rhinitis, asthma, and allergic conjunctivitis. Cardiometabolic comorbidity was defined as the presence of two or more of the following: hypertension, type 2 diabetes mellitus, or dyslipidemia. This definition was selected based on the three core components of metabolic syndrome, as these variables were the most consistently documented cardiovascular risk factors in our retrospective EMR. Smoking status was excluded from the cardiometabolic comorbidity definition due to inconsistent EMR documentation.

### 2.3. Safety Monitoring

Throughout the study, treatment-emergent adverse events (TEAEs), serious adverse events (SAEs; severity graded per ICH E2A), and adverse events of special interest (AESIs) were documented. AESIs specifically included MACE, VTE, serious infections, herpesvirus infections, and malignancy. Laboratory monitoring consisted of hematology, liver function, renal function, lipid, and creatine kinase evaluations, which were conducted at baseline and every 3 to 6 months thereafter.

### 2.4. Statistical Analysis

Continuous variables were summarized as medians with interquartile ranges (IQRs) and categorical variables as counts with percentages. Drug persistence was estimated with the Kaplan–Meier method using a 36-month cutoff; patients still on treatment at the last follow-up or at the data cutoff date (July 2026) were censored, and 95% confidence intervals (CIs) were obtained from the Kaplan–Meier estimator. Stratified analyses grouped patients by baseline psychotropic medication use, CCI (dichotomized at the cohort median of 5), polypharmacy, and atopic comorbidities; survival curves were compared with the log-rank test.

Eight candidate predictors of persistence—age, sex, baseline EASI, CCI, polypharmacy, atopic comorbidities, Week 4 itch response, and baseline psychotropic medication use—were screened by univariate Cox regression. Variables with *p* < 0.10 were taken forward. Guided by prior clinical hypotheses and maintaining an events per variable ratio ≥ 10 [12], a multivariate Cox model containing Week 4 itch response and baseline psychotropic medication use was fitted. Hazard ratios (HRs) and 95% CIs were computed. The proportional hazards assumption was checked with Schoenfeld residuals, and multicollinearity was assessed with variance inflation factors. All tests were two-sided, with *p* < 0.05 considered significant. Analyses were performed in SPSS (version 26.0; IBM Corp., Armonk, NY, USA). Figures were produced with GraphPad Prism (version 11,GraphPad Software, Boston, MA, USA), and the forest plot was generated with Python (version 3.10) using the Matplotlib library (version 3.7.1). Given the exploratory and retrospective nature of this study, which used a consecutive convenience sample, a formal pre-study sample size or sensitivity/power calculation was not performed.

## 3. Results

### 3.1. Baseline Characteristics

Sixty-two patients were enrolled (Table 1). The median age was 70.0 years (IQR 67.0–76.0); 47 (75.8%) were male. Baseline disease was severe: median EASI 34.5 (IQR 23.0–45.0), median pruritus NRS 8.0 (IQR 7.0–8.0). Comorbidities were prevalent: 66.1% carried ≥2 cardiometabolic conditions, median CCI was 5.0 (IQR 4.0–6.0), polypharmacy was recorded in 56.5%, and atopic comorbidities in 58.1%. Baseline psychotropic medication use was documented in 29 patients (46.8%). Prior dupilumab failure rate was 80.6%; 22.6% had previously received other oral JAK inhibitors. Median follow-up was 12.5 months (IQR 5.0–24.0; maximum 51.7 months; the primary analysis used a 36-month data cutoff).

### 3.2. Efficacy

A Week 4 itch response was achieved by 70.8% of patients. EASI 75 rose from 75.0% at Week 12 to 88.0% at Week 24; EASI 90 rose from 47.2% to 60.0%. IGA 0/1 increased from 44.4% at Week 12 to 64.0% at Week 24 (Table 2). Table 2 presents observed-case analysis using data from patients who had available assessments at the respective time points. Patients who discontinued before Week 12 or 24 were not included in the denominator for that time point.

### 3.3. Safety

No patient discontinued upadacitinib due to adverse events. TEAEs occurred in 54.8% (34/62) and SAEs in 8.1% (5/62). Herpesvirus infection was recorded in 21.0% (13/62). Clinically, these were primarily diagnosed as reactivations of herpes zoster or herpes simplex, rather than new primary infections. It should be noted that reactivations are common in the elderly population. One severe infection, a skin and soft tissue abscess, was resolved with drainage without treatment interruption. Laboratory abnormalities were detected in 25.8% (16/62); all events were mild and asymptomatic, and none required treatment discontinuation. Neither MACE nor VTE was observed (Table 3). Adverse events were recorded during each patient’s entire available follow-up period, including those with extended observation beyond 36 months.

### 3.4. Drug Persistence

**Figure 1:**
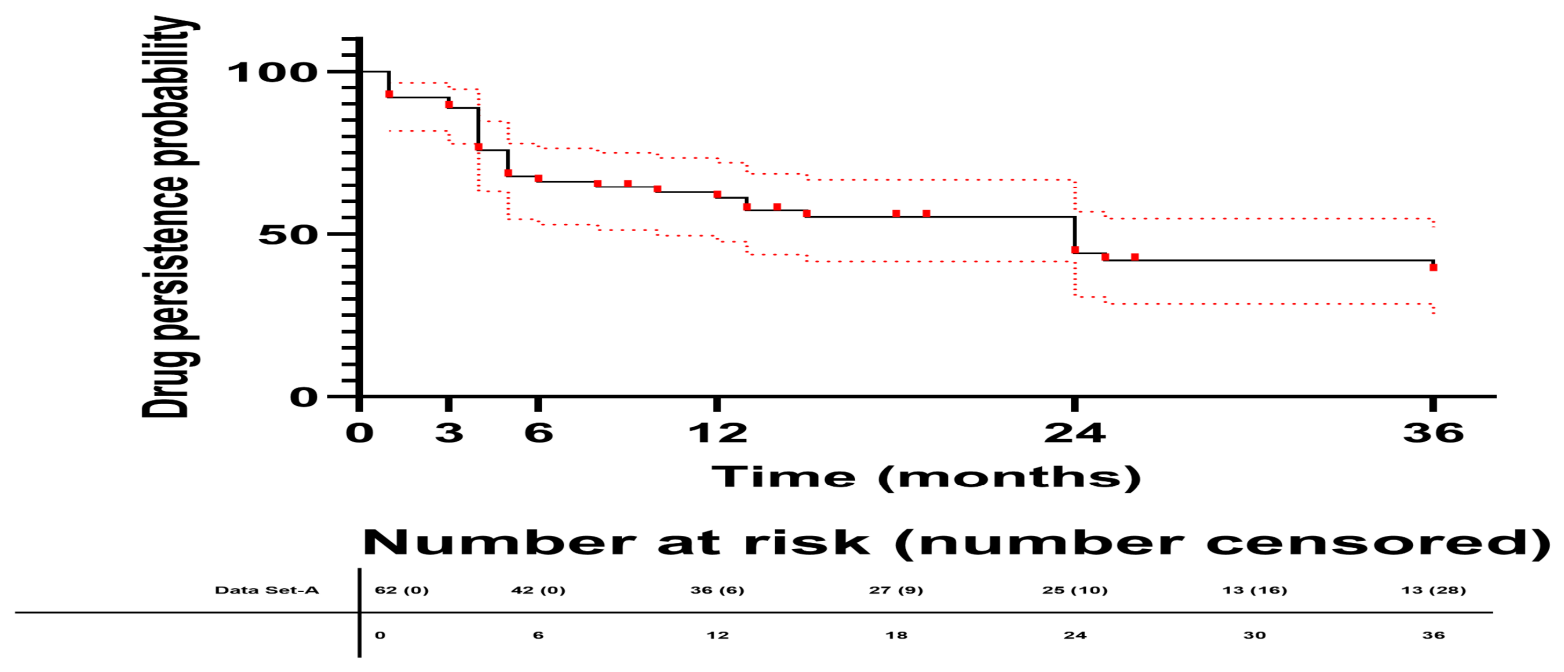
Kaplan–Meier curve of drug persistence of upadacitinib in patients aged ≥ 65 years with moderate-to-severe atopic dermatitis through 36 months of follow-up. Solid line: estimated persistence; dashed lines: 95% *CI*; tick marks: censored data. Numbers at risk at 0, 3, 6, 12, 24, and 36 months are shown below the x-axis.

Kaplan–Meier cumulative persistence at the 36-month cutoff was 88.7% at Month 3, 66.1% at Month 6, 61.1% at Month 12, 44.2% at Month 24, and 38.7% at Month 36 (Figure 1). The steepest portion of the curve occurred between Months 3 and 6, corresponding to an absolute decline of 22.6% and an average monthly drop of 7.53% (Table 4).

### 3.5. Stratified Persistence Analyses

**Figure 2:**
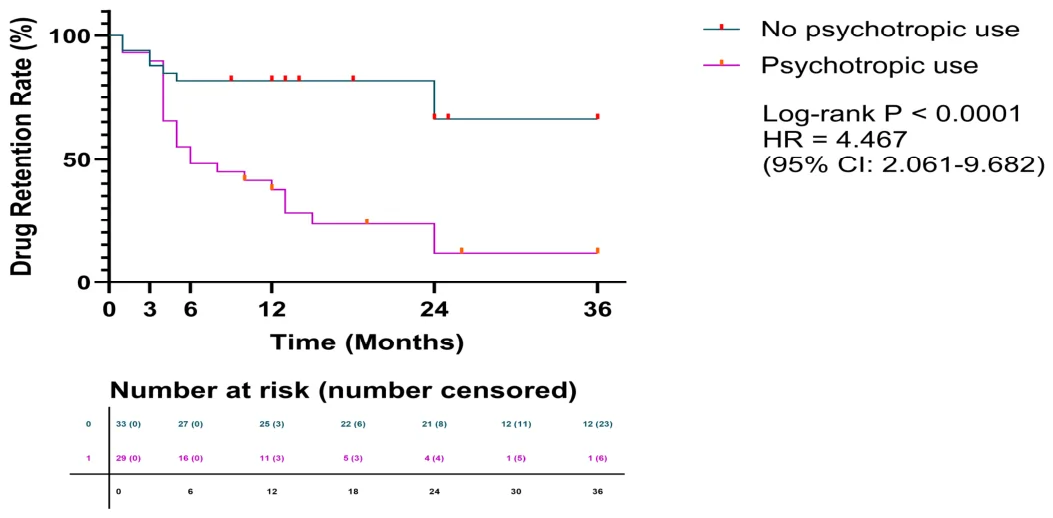
Kaplan–Meier curves of drug persistence stratified by baseline psychotropic medication use. Patients with baseline psychotropic medication use had significantly shorter drug persistence compared to those without (log-rank *p* < 0.0001; *HR* = 4.467, 95% *CI*: 2.061–9.682). Shaded areas indicate 95% confidence intervals. Tick marks indicate censored data. Numbers at risk are shown below the x-axis at 6-month intervals.

Persistence was consistently lower among patients who used psychotropic medications at baseline than among non-users (log-rank *p* < 0.0001; Figure 2), with the survival curves beginning to separate at approximately Month 3. In contrast, stratifying by CCI (≥5 vs. <5), polypharmacy, or atopic comorbidities produced overlapping curves and no statistically significant differences (Supplementary Figures S1–S3).

### 3.6. Predictors of Discontinuation

Univariate Cox regression identified Week 4 itch response (*HR* 0.151, 95% *CI* 0.070–0.323, *p* < 0.001) and baseline psychotropic medication use (*HR* 4.467, 95% *CI* 2.061–9.682, *p* < 0.001) as the only variables associated with persistence. Age, sex, baseline EASI, CCI, polypharmacy, and atopic comorbidities did not approach significance (Supplementary Table S1).

**Figure 3:**
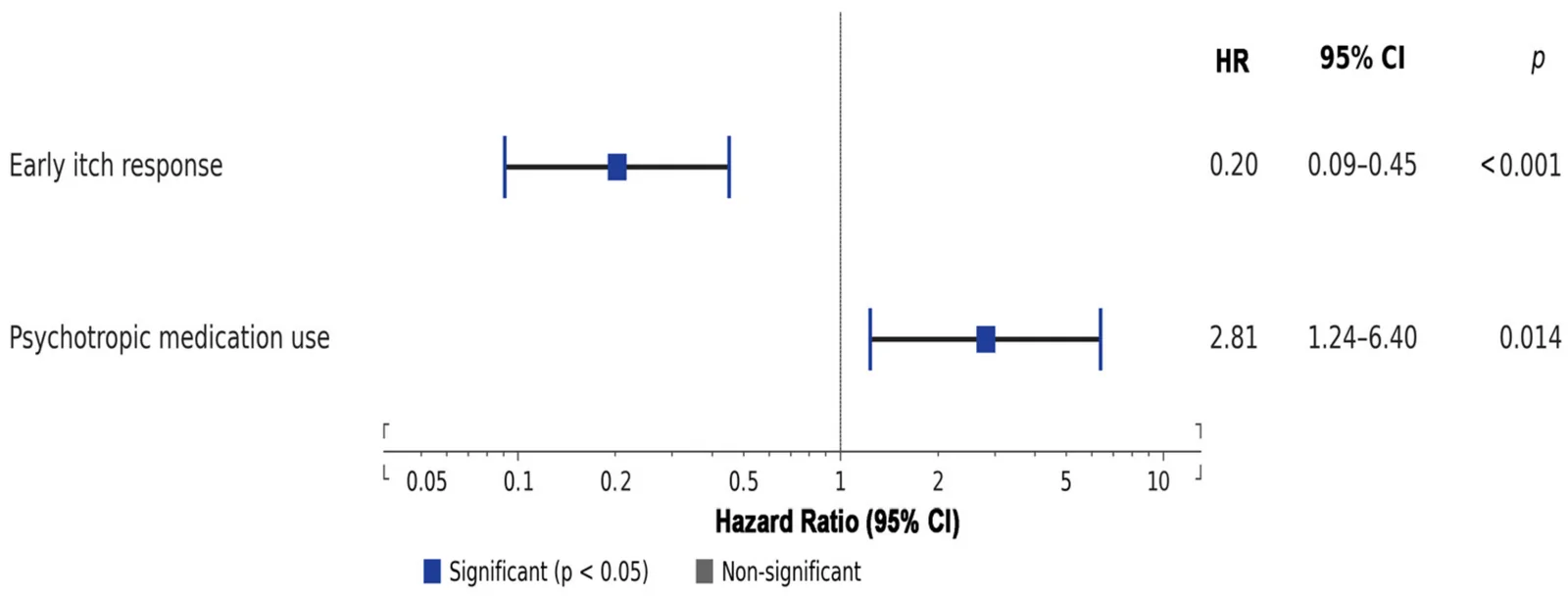
Forest plot of multivariate Cox regression analysis for factors associated with drug persistence. Hazard ratios (*HRs*) and 95% confidence intervals (*CIs*) are plotted on a logarithmic scale. Squares represent point estimates; horizontal lines represent 95% *CIs*. The vertical dashed line at *HR* = 1 indicates no effect. Week 4 itch response was independently associated with prolonged drug persistence (*HR* = 0.203, 95% *CI*: 0.091–0.452, *p* < 0.001), whereas baseline psychotropic drug use was independently associated with shorter drug persistence (*HR* = 2.814, 95% *CI*: 1.237–6.403, *p* = 0.014).

When both variables were entered into a multivariate Cox model (Table 5), each retained independent prognostic value. Week 4 itch response was linked to longer persistence (*HR* 0.203, *95% CI* 0.091–0.452, *p* < 0.001), whereas baseline psychotropic medication use conferred a higher risk of discontinuation (*HR* 2.814, 95% *CI* 1.237–6.403, *p* = 0.014) (Figure 3). The overall model was significant (*χ*^2^ = 32.930, *df* = 2, *p* < 0.001), the proportional hazards assumption was satisfied, and variance inflation factors remained below 2.0.

### 3.7. Reasons for Discontinuation and Dose Management

Of the 33 patients who stopped upadacitinib, disease remission was the most common reason (10, 30.3%). Psychological distress or decreased motivation accounted for 24.2% (8), followed by inadequate efficacy (5, 15.2%), safety concerns (3, 9.1%), and financial constraints (4, 12.1%) (Supplementary Table S2). A total of 21 patients (45.7%) extended their dosing interval after achieving disease control; baseline characteristics did not differ materially between the standard dose and extended interval groups (Supplementary Table S3).

## 4. Discussion

This 36-month real-world study of upadacitinib in elderly patients with moderate-to-severe AD addressed three interrelated questions: long-term effectiveness and safety in a multimorbid population, the trajectory of drug persistence, and the determinants of treatment discontinuation. The findings form a coherent narrative with direct clinical implications.

### 4.1. Robust Efficacy Despite Challenging Baseline Characteristics

The Week 24 EASI 75 response of 85.5% and IGA 0/1 rate of 62.9% align closely with phase III data [2,3] and long-term extension results [13,14], yet the context is fundamentally different: the median baseline EASI exceeded 34, over 80% of patients had failed dupilumab, and the median CCI stood at five. This confirms that advanced age, comorbidity, and prior biologic exposure do not attenuate upadacitinib’s skin clearance efficacy. The finding directly addresses a common clinical question: whether a 75-year-old with multimorbidity and prior dupilumab failure would respond similarly to trial populations. The answer is affirmative. Equally important, over 60% of patients achieved meaningful itch relief by Week 4, an early signal that proved decisive for long-term adherence.

### 4.2. Reassuring Safety Profile in a Multimorbid Population

Over 36 months, no patient discontinued upadacitinib due to an adverse event, a finding consistent with the Italian IL AD study of JAK inhibitors in patients aged over 60 years [4]. No MACE or VTE were observed, which contrasts with the ORAL Surveillance trial but is explained by the fundamentally different cardiovascular risk architecture of the two populations: active RA with enriched cardiovascular risk factors versus ambulatory AD patients without acute cardiovascular disease [8]. Herpesvirus reactivation was observed in 21.0% of patients. This rate is higher than the 2–4% reported in phase III trials [3,13] and the 5–10% described in shorter-term real-world cohorts of older AD patients [4,5]. The extended follow-up of our study, the advanced age of the cohort, and the low herpes zoster vaccination coverage in Chinese older adults may all contribute to this difference. SAEs were uncommon and resolved without sequelae. Laboratory abnormalities were mild and asymptomatic, requiring no treatment interruption. A single case of localized prostate cancer, diagnosed two months after drug initiation, could not be causally linked to treatment given the brief latency and background incidence.

### 4.3. Drug Persistence and the 3–6-Month Critical Window

Over the 36-month follow-up period, drug persistence in this cohort declined progressively: 88.7% at Month 3, 66.1% at Month 6, 61.1% at Month 12, 44.2% at Month 24, and 38.7% at Month 36 (Figure 1). Notably, despite the excellent efficacy observed in our cohort (e.g., 88.0% achieving EASI 75 at Week 24 among patients who remained on treatment), the overall 36-month drug persistence was only 38.7%. This divergence suggests that factors beyond efficacy, including patient adherence and psychological vulnerability, may be important contributors to long-term retention. In the Italian multicenter study by Pezzolo et al., which enrolled younger patients (mean age 38.6 years), the 1-year persistence rate was 91.5% [6], substantially higher than our 61.1% at the same time point. By contrast, the Dutch cohort reported by Schlösser et al. (18-month persistence 48.4%) [15], and the Swedish SwedAD registry (2-year persistence 46.0% for upadacitinib) [16] all showed estimates much closer to ours. These comparisons suggest that persistence rates tend to be lower in older and more complex populations, although the precise drivers of these differences remain to be determined.

The 3-6 month period represents a critical window for drug persistence, characterized by the highest risk of treatment discontinuation rather than a peak in treatment-response. Beyond these cross-study comparisons, the interval analysis of our own data revealed a temporal pattern that we consider clinically more important than the absolute persistence rates themselves. The steepest decline occurred between Month 3 and Month 6, with an absolute reduction of 22.6% (88.7% to 66.1%) over a single quarter, more than double the attrition observed in any other interval (16.9% from Month 12 to 24, and only 5.5% from Month 24 to 36). Most previous studies have reported persistence at isolated time points; our findings suggest that this practice may obscure a highly time-specific pattern of early treatment discontinuation.

Why would patients who had already tolerated the drug and achieved an initial response (Week 24 EASI 75 88.0%) cluster their discontinuation within the 3- to 6-month window? This pattern is unlikely to be explained by primary efficacy failure or tolerability issues alone, prompting us to examine baseline characteristics and early treatment response as potential drivers of persistence.

### 4.4. Psychotropic Medication Use as the Sole Discriminator

The overall KM curve (Figure 1) revealed the 3- to 6-month period as the phase of steepest attrition, but did not specify which patients were most vulnerable. To identify patients at high risk of dropping out during this window, we performed stratified Kaplan–Meier analyses on four prespecified clinically relevant variables (CCI grouped by the cohort median, polypharmacy, psychotropic medication use, and atopic comorbidity) [9,17,18].

Stratified analyses revealed that only baseline psychotropic medication use significantly distinguished persistence trajectories, with the two curves diverging from Month 3 onward. Discontinuation among psychotropic users was concentrated within the 3 to 6-month window: during the first 6 months, the attrition rate was 65.5% in the psychotropic group versus 21.2% in the non-psychotropic group. By Month 36, persistence was 6.9% versus 33.3%, respectively—a nearly five-fold difference. The remaining variables showed no discriminatory value.

This finding suggests that the 3- to 6-month attrition peak observed in the overall KM curve was largely driven by patients with baseline psychotropic medication use. Psychological vulnerability, rather than somatic disease burden or medication count, emerged as the predominant driver of early discontinuation. This observation aligns with existing evidence: adults with AD have a 2- to 3-fold higher risk of depression and anxiety [17], AD is independently associated with elevated depression risk [18], and shared genetic susceptibility loci have been identified between AD and major depressive disorder [19].

Stratified analyses, however, can only determine whether a variable distinguishes persistence trajectories; they cannot establish their independent effects after adjusting for confounders. According to our statistical plan, only variables with *p* < 0.10 in the univariate analysis (Supplementary Table S1) were entered into the multivariate model. Consequently, the final model included only Week 4 itch response and baseline psychotropic medication use, as they were the only variables meeting this entry criterion.

### 4.5. Independent Predictors of Discontinuation: Multivariate Cox Regression

Multivariable Cox regression analysis confirmed that early itch response (Week 4 NRS reduction ≥ 4) was associated with an approximately 80% reduction in discontinuation risk. Baseline psychotropic medication use, in contrast, conferred a nearly 3-fold increase in discontinuation risk. Of note, the univariate HR for psychotropic medication use was 4.467; the attenuation after adjustment for Week 4 itch response suggests that part of its effect is mediated through early symptom improvement, while a substantial independent component remains.

The two predictors converge at the 3- to 6-month window. Early itch relief provides direct experiential evidence of therapeutic benefit. Hagino et al. reported that among patients treated with upadacitinib, those achieving PP NRS ≤ 1 at Week 2 attained a 42.9% EASI 100 rate at Week 24, versus 1.4% in non-responders [20]. This suggests that early symptom improvement not only signals short-term benefit but also predicts long-term clearance, thereby reinforcing adherence during the consolidation phase.

Conversely, baseline psychotropic medication use reflects greater psychological vulnerability. In a propensity-matched cohort study of 447,821 pairs, Szepietowska et al. found that AD patients had approximately three-fold higher utilization of psychological services (RR ≈ 3.0) and significantly elevated psychiatric medication use (RR ≈ 1.35) [21]. Patients on psychotropic medications carry a disproportionate burden of depression, anxiety, and stress-related disorders—conditions known to undermine treatment adherence. During the 3- to 6-month consolidation period, this vulnerability heightens sensitivity to safety concerns and reduces tolerance of transient side effects, gradually tipping the balance toward discontinuation.

### 4.6. Interpretation of Discontinuation and Dose Management

Among the 33 patients who discontinued treatment, disease remission was the most common reason (10, 30.3%), followed by psychological distress (8, 24.2%), ahead of inadequate efficacy (5, 15.2%), financial constraints (4, 12.1%), and safety concerns (3, 9.1%) (Supplementary Table S2). The prominence of psychological distress as the second leading cause was consistent with the Cox regression findings, where psychotropic medication use emerged as an independent predictor of discontinuation. The concordance between statistically identified risks and patient-reported reasons suggests that psychological factors are not merely predictive variables but actual drivers of discontinuation behavior.

Dose interval extension was adopted across diverse baseline profiles, with no significant differences in key baseline characteristics between the standard-dose and extended-interval groups (Supplementary Table S3), suggesting that de-escalation strategies are not limited to patients with milder disease.

These findings set the stage for the front-loaded management pathway described below.

### 4.7. Translating Findings into Practice: A Front-Loaded Management Pathway

The findings from this study point to a single clinical action: shifting the focus of management forward to the first three months of treatment, with proactive identification and intervention for high-risk patients during the 3- to 6-month window. At baseline, psychotropic medication use can be efficiently identified through routine medication reconciliation. At Week 4, itch response serves as the patient’s initial efficacy experience, and non-responders warrant closer attention. The Month 3 visit becomes the critical checkpoint, integrating itch reassessment with brief mental health screening, allowing intervention before safety concerns solidify or psychological distress accumulates. For patients achieving disease control, dose interval extension may be discussed as an individualized de-escalation strategy. For those with both baseline psychotropic use and Week 4 nonresponse, intensified follow-up every 4 6 weeks through Month 6 is recommended.

This pathway requires no additional resources—only three deliberate actions embedded into routine visits: review the medication list, reassess itch, and screen mental health. These small, systematic steps may determine whether a vulnerable elderly patient remains on effective therapy or discontinues prematurely. Future prospective, multicenter studies utilizing validated psychometric scales are warranted to confirm the predictive value of early itch response and psychotropic medication use in elderly AD patients.

### 4.8. Limitations

Several limitations should be noted. First, the single-center, retrospective nature, the modest sample size (62 patients), and the high proportion of dupilumab-experienced individuals (80.6%)—indicative of a treatment-refractory, high burden population—limit the generalizability of the findings; results should be extrapolated with caution to biologic naïve or less severely affected older AD cohorts. Second, psychological status was inferred from prescription records rather than measured with validated instruments, which may have misclassified untreated distress or included medications prescribed for non-psychiatric reasons. Third, the number of patients still remaining on treatment and under active follow-up beyond 24 months was small (*n* = 13), so the 36-month persistence estimate should be interpreted with appropriate caution. Fourth, as an exploratory real-world analysis, these findings should be considered hypothesis-generating. The identified predictors require confirmation in larger, multicenter prospective cohorts before they can be integrated into routine clinical decision-making. Fifth, given the exploratory nature of this retrospective study using a consecutive sample, no formal pre-study sample size or sensitivity/power calculation was performed.

## 5. Conclusions

Upadacitinib provided sustained efficacy and acceptable long-term safety over 36 months in elderly patients with moderate-to-severe AD, with no AE-related discontinuations and no MACE or VTE events. Drug persistence fell most sharply between Months 3 and 6, a period during which approximately one-quarter of patients stopped treatment despite having already benefited from it. Two clinical indicators, both readily available in routine practice—Week 4 itch response and baseline psychotropic medication use—emerged as independent determinants of persistence. These findings argue for moving the clinical focus forward: early identification of vulnerable patients, combined with intentional follow-up during the 3–6 month consolidation window, may keep more patients on a treatment that is working.

## Figures and Tables

**Table 1:** Baseline demographic and clinical characteristics (*n* = 62).

Variable	Value
Demographics and disease characteristics	
Age, years, median (IQR)	70.0 (67.0–76.0)
Male sex, *n* (%)	47 (75.8)
AD duration, years, median (IQR)	7.0 (4.0–15.0)
Baseline EASI, median (IQR)	34.5 (23.0–45.0)
Baseline pruritus NRS, median (IQR)	8.0 (7.0–8.0)
Comorbidity burden	
Hypertension, *n* (%)	39 (62.9)
Type 2 diabetes mellitus, *n* (%)	25 (40.3)
Dyslipidemia, *n* (%)	35 (56.5)
≥2 cardiometabolic comorbidities *, *n* (%)	41 (66.1)
Atopic comorbidities †, *n* (%)	36 (58.1)
CCI, median (IQR)	5.0 (4.0–6.0)
Polypharmacy (≥5 oral medications), *n* (%)	35 (56.5)
Baseline psychotropic medication use ‡, *n* (%)	29 (46.8)
Treatment history	
Prior dupilumab use, *n* (%)	50 (80.6)
Prior other JAK inhibitor use, *n* (%)	14 (22.6)
Follow-up	
Follow-up duration, months, median (IQR)	12.5 (5.0–24.0)

* Cardiometabolic comorbidities: type 2 diabetes mellitus, ≥2 of hypertension, or dyslipidemia. † Atopic comorbidities: allergic rhinitis, asthma, or allergic conjunctivitis. ‡ Psychotropic medications: antidepressants, anxiolytics, or hypnotics.

**Table 2:** Efficacy outcomes (observed-case analysis).

Outcome	*n*/*N* (%)
Week 4	
Week 4 itch response (≥4-point NRS reduction), *n* (%)	34/48 (70.8)
Week 12	
EASI 75, *n* (%)	27/36 (75.0)
EASI 90, *n* (%)	17/36 (47.2)
IGA 0/1, *n* (%)	16/36 (44.4)
Week 24	
EASI 75, *n* (%)	22/25 (88.0)
EASI 90, *n* (%)	15/25 (60.0)
IGA 0/1, *n* (%)	16/25 (64.0)

EASI 75/90: A ≥75%/≥90% reduction in Eczema Area and Severity Index from baseline. IGA 0/1: Investigator‘s Global Assessment score of 0 (clear) or 1 (almost clear). Week 4 itch response was defined as a ≥4-point reduction in the peak pruritus Numerical Rating Scale (NRS) score from baseline at week 4. Table 2 presents observed-case (OC) analysis using data from patients who had available assessments at the respective time points. The denominators (*N*) represent the actual number of patients still on treatment and actively followed up at each time point (Week 4: *N* = 48; Week 12: *N* = 36; Week 24: *N* = 25). Patients who discontinued before these time points were not included in the denominator.

**Table 3:** Safety outcomes during the observation period (*n* = 62).

Event	*n* (%)
Any TEAE	34 (54.8)
SAE	5 (8.1)
Discontinuation due to AE	0 (0.0)
Severe infection	1 (1.6)
Herpesvirus infection	13 (21.0)
MACE	0 (0.0)
VTE	0 (0.0)
Malignancy *	1 (1.6)
Laboratory abnormalities †	16 (25.8)

TEAE: treatment-emergent adverse event; SAE: serious adverse event; MACE: major adverse cardiovascular event; VTE: venous thromboembolism. * One case of localized prostate adenocarcinoma in a 77-year-old male with stable post-surgical status; no causal link to upadacitinib was established. † Included creatine kinase elevation (*n* = 4), transaminase elevation (*n* = 6), leukopenia/neutropenia (*n* = 4), dyslipidemia (*n* = 2), and proteinuria (*n* = 1). All were asymptomatic and were manageable with dose adjustment or observation.

**Table 4:** Drug persistence rates and slope of decline by time interval (*n* = 62).

Time Interval (mo)	Duration (mo)	*N* at Risk (Start)	Persistence at Start (%)	Persistence at End (%)	Absolute Decline (%)	Average Monthly Decline (%/mo)
0–3	3	62	100.0	88.7	11.3	3.77
**3–6**	**3**	**54**	**88.7**	**66.1**	**22.6**	**7.53**
6–12	6	36	66.1	61.1	5.0	0.83
12–24	12	27	61.1	44.2	16.9	1.41
24–36	12	13	44.2	38.7	5.5	0.46

Bold indicates the critical window (3–6 months) with the steepest absolute decline and highest average monthly decline. All values were derived from Kaplan–Meier life-table output (*N* = 62). 36-month cumulative persistence = 38.7%.

**Table 5:** Multivariate Cox regression analysis of factors related to drug persistence.

Variable	*β*	*SE*	*Wald χ* ^2^	*HR* (95% *CI*)	*p* Value
Week 4 itch response (>4-point NRS reduction)	−1.594	0.408	15.267	0.203 (0.091–0.452)	<0.001
Baseline psychotropic medication use	1.035	0.419	6.085	2.814 (1.237–6.403)	0.014

Model *χ*^2^ = 32.930 (*df* = 2, *p* < 0.001); −2 Log Likelihood = 215.436. The proportional hazards assumption was satisfied (Schoenfeld residuals, *p* > 0.05), and variance inflation factors were <2.0 for both variables. The events per variable (EPV) ratio was 16.5. *HR*: hazard ratio; *CI*: confidence interval; NRS: Numerical Rating Scale.

## Data Availability

The datasets generated and/or analyzed during the current study are available from the corresponding authors on reasonable request.

## Supplementary Materials

The following supporting information can be downloaded at: https://www.mdpi.com/article/10.3390/jcm15176593/s1, Table S1. Univariate Cox regression analysis of candidate predictors of discontinuation. Table S2. Categorized reasons for upadacitinib discontinuation. Table S3. Comparison of baseline characteristics between standard dose and extended interval upadacitinib groups. Figure S1. Stratified Kaplan–Meier curves by Charlson Comorbidity Index (dichotomized at the cohort median of 5). Figure S2. Stratified Kaplan–Meier curves by polypharmacy. Figure S3. Stratified Kaplan–Meier curves by atopic comorbidities.

## Abbreviations

The following abbreviations are used in this manuscript:

AD, atopic dermatitis; AE, adverse event; AESI, adverse event of special interest; CCI, Charlson Comorbidity Index; CI, confidence interval; EASI, Eczema Area and Severity Index; EPV, events per variable; FDA, Food and Drug Administration; HR, hazard ratio; IGA, Investigator’s Global Assessment; IQR, interquartile range; JAK, Janus kinase; KM, Kaplan–Meier; MACE, major adverse cardiovascular event; NRS, Numerical Rating Scale; SAE, serious adverse event; TEAE, treatment-emergent adverse event; T2T, treat-to-target; VTE, venous thromboembolism.
