# Supplementary material for: Real-World Effectiveness, Safety, and 36-Month Persistence of Upadacitinib in Elderly Patients with Moderate-to-Severe Atopic Dermatitis: The 3–6 Month Critical Window and the Role of Early Itch Response and Psychotropic Medication Use
Source: J Clin Med. 2026 Aug 26;15(17):6593. doi: 10.3390/jcm15176593 (PMC13566308; doi:10.3390/jcm15176593)
Supplement: Supplementary file 1 [file jcm-15-06593-s001.zip › Supplementary Table S1.pdf]

Supplementary Table S1. Univariate Cox regression analysis of candidate predictors of discontinuation.

Variable	HR (95% CI)	P value
Age (per 1-year increase)	1.003 (0.948–1.062)	0.913
Male sex (vs. female)	1.330 (0.577–3.066)	0.503
Baseline EASI (per 1-point increase)	0.976 (0.947–1.006)	0.117
CCI (per 1-point increase)	0.885 (0.705–1.111)	0.293
Polypharmacy (≥5 medications vs. <5)	1.126 (0.560–2.264)	0.739
Atopic comorbidities (yes vs. no)	0.871 (0.437–1.739)	0.696
Baseline psychotropic medication use (yes vs. no)	4.467 (2.061–9.682)	<0.001
Week 4 itch response (yes vs. no)	0.151 (0.070–0.323)	<0.001

Each variable was examined in a separate univariate Cox regression model.
HR: hazard ratio; CI: confidence interval; EASI: Eczema Area and Severity Index; CCI: Charlson Comorbidity Index.
