# Supplementary material for: Real-World Effectiveness, Safety, and 36-Month Persistence of Upadacitinib in Elderly Patients with Moderate-to-Severe Atopic Dermatitis: The 3–6 Month Critical Window and the Role of Early Itch Response and Psychotropic Medication Use
Source: J Clin Med. 2026 Aug 26;15(17):6593. doi: 10.3390/jcm15176593 (PMC13566308; doi:10.3390/jcm15176593)
Supplement: Supplementary file 1 [file jcm-15-06593-s001.zip › Supplementary Table S2.pdf]

Supplementary Table S2. Categorized reasons for upadacitinib discontinuation.

Reason	n (%)
Disease remission (physician-directed or patient-initiated)	10 (30.3)
Inadequate efficacy	5 (15.2)
Safety concerns (including black-box warning)	3 (9.1)
Financial constraints	4 (12.1)
Psychological distress / decreased treatment motivation	8 (24.2)
Other (intercurrent hospitalization, loss to follow-up, unknown)	3 (9.1)
Total discontinuations	33 (100)

Percentages are based on the 33 patients who discontinued upadacitinib. Reasons were assigned in a mutually exclusive manner; for each patient, the single most clinically relevant reason was abstracted from the medical record.
