# Supplementary material for: Real-World Effectiveness, Safety, and 36-Month Persistence of Upadacitinib in Elderly Patients with Moderate-to-Severe Atopic Dermatitis: The 3–6 Month Critical Window and the Role of Early Itch Response and Psychotropic Medication Use
Source: J Clin Med. 2026 Aug 26;15(17):6593. doi: 10.3390/jcm15176593 (PMC13566308; doi:10.3390/jcm15176593)
Supplement: Supplementary file 1 [file jcm-15-06593-s001.zip › Supplementary Table S3.pdf]

Supplementary Table S3. Comparison of baseline characteristics between standard-dose and extended-interval upadacitinib groups.

Characteristic	Standard-dose (n=36)	Extended-interval (n=26)	P value
**Demographics**			
Age, years, median (IQR)	70.0 (67.0–75.0)	69.5 (66.0–78.0)	0.612
Male sex, n (%)	28 (77.8)	19 (73.1)	0.767
AD duration, years, median (IQR)	7.0 (4.0–10.0)	7.0 (4.0–15.0)	0.473
Baseline EASI, median (IQR)	28.0 (23.0–45.0)	36.0 (26.0–46.0)	0.329
Baseline pruritus NRS, median (IQR)	8.0 (7.0–8.0)	8.0 (7.0–8.0)	0.325
**Comorbidity burden**			
CCI, median (IQR)	5.0 (4.0–6.0)	5.0 (4.0–6.0)	0.815
Hypertension, n (%)	21 (58.3)	17 (65.4)	0.608
Type 2 diabetes mellitus, n (%)	13 (36.1)	12 (46.2)	0.449
Dyslipidemia, n (%)	20 (55.6)	15 (57.7)	0.999
⩾2 cardiometabolic comorbidities, n (%)	18 (50.0)	19 (73.1)	0.075

Characteristic	Standard-dose (n=36)	Extended-interval (n=26)	P value
Atopic comorbidities, n (%)	22 (61.1)	15 (57.7)	0.802
Polypharmacy (≥5 medications), n (%)	17 (47.2)	18 (69.2)	0.121
Baseline psychotropic medication use, n (%)	17 (47.2)	12 (46.2)	0.999
**Treatment history**			
Prior dupilumab use, n (%)	30 (83.3)	20 (76.9)	0.545
Prior JAK inhibitor use, n (%)	8 (22.2)	7 (26.9)	0.769
**Follow-up**			
Follow-up duration, months, median (IQR)	24.0 (12.0–36.0)	12.0 (5.0–36.0)	0.096

Continuous variables were compared with the Mann–Whitney U test and are shown as median (IQR). Categorical variables were compared with Fisher's exact test and are shown as n (%). All P values are two-sided.

Standard-dose: upadacitinib 15 mg once daily. Extended-interval: upadacitinib 15 mg every 2 days or less frequently (e.g., every 2, 3, 4, or 6 days). Patients with documented dose intervals were included irrespective of treatment status; those without a recorded interval were excluded.
